# The CEIP-Framework – From Reaction to Prevention in Health in All and for Policies

**DOI:** 10.3389/phrs.2025.1608225

**Published:** 2025-03-21

**Authors:** Julia Nadine Doetsch, Ponciano Oliveira, Henrique Barros

**Affiliations:** ^1^ Epidemiological Research Unit (EPIunit), Institute of Public Health of the University of Porto (ISPUP), Porto, Portugal; ^2^ Laboratório para a Investigação Integrativa e Translacional em Saúde Populacional (ITR), Porto, Portugal; ^3^ Faculty of Law, University of Porto, Porto, Portugal

**Keywords:** health equity, non-communicable chronic diseases (NCDs), health in all policies, political determinants of health, CEIP framework

## Introduction

Health in All Policies (HiAP) assumes policy synergies. Health outcomes are influenced by political, social, economic, and environmental determinants defined outside of traditional health system institutions. HiAP integrates health considerations across multiple sectors to increase population health capital, and by addressing social determinants of health (SDH), it might contribute to decreasing health inequities. The World Health Organization recently called for the complementary Health for All Policies strategy to enable positive outcomes across sectors and allow a broader policy integration [1, 2].

HiAP, *inter alia*, highlights the role of political determinants—how power, resources, and relationships shape social conditions and maintain health inequalities [3]. Therefore, political determinants must be funneled through a health equity framework to address inequalities.

Legal support does not guarantee the successful implementation of HiAP. Portugal (see reference 1 in Supplementary Data Sheet 1) might be an example: a rich legal trajectory resulted in HiAP’s inconsistent implementation due to industry interests and the inability to overcome political contradictions.

In Portugal, major difficulties derive from weak cross-sectoral collaborations due to: limited resource allocation [4], unused data-sharing opportunities [5], and fragmented policy efforts [6]. Reactive strategies that prioritize short-term economic gains over preventive health measures, combined with inadequate governance structures for cross-sectoral alignment of public health goals, hamper HiAP [6, 7].

Political and industry lobbying and cultural acceptance make it difficult to enact laws that change behavior [4]. Lack of institutional support in trade, finance, and culture, aggravates these challenges (e.g., wine, a major national export).

Despite Portugal’s legislative efforts to control tobacco, alcohol, gambling and sugar, these remain pressing public health issues:

High youth smoking rates indicate that Tobacco regulations (see reference 2 in Supplementary Data Sheet 1), imposing significant restrictions on smoking in public spaces and requiring the display of health warnings on tobacco products (see reference 3 in Supplementary Data Sheet 1), are not sufficient to diminish youth smoking [4].

The implementation of alcohol regulations (see reference 4 in Supplementary Data Sheet 1), which prohibit the sale of alcoholic beverages to minors, has proven to be inconsistent in protecting young people [8].

The Integrated Strategy for the Promotion of Healthy Eating, yet to be evaluated, addresses sugar consumption through the sugar tax (see reference 5 in Supplementary Data Sheet 1).

There is an increasing accessibility and prevalence of online gambling raising significant public health concerns due to associated harms [9]. Despite the goals of regulating, it and simultaneously generating tax revenues (see reference 6 in Supplementary Data Sheet 1), the continued presence of media advertisements might need additional regulations for the above-mentioned.

## The CEIP-Framework–From Reaction to Prevention

**FIGURE 1:**
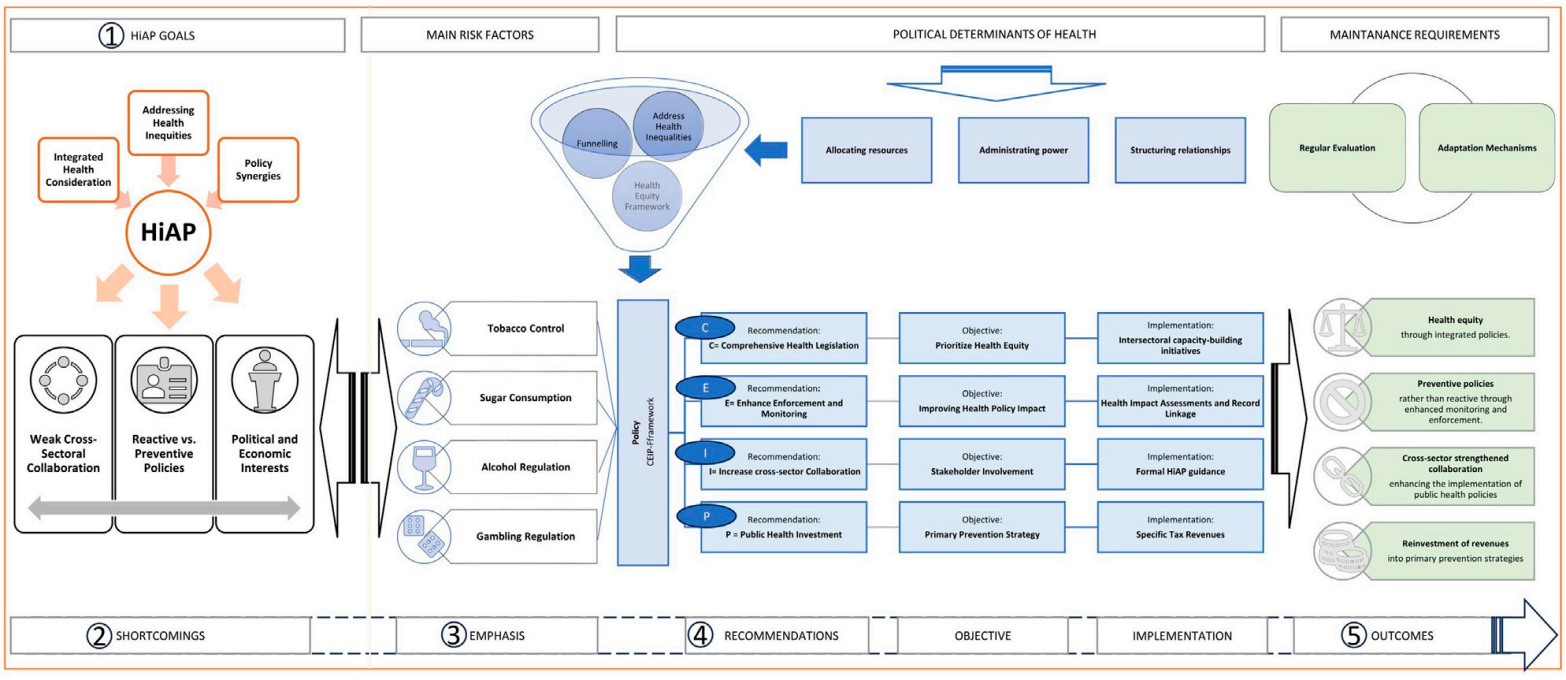
The CEIP-framework - from reaction to prevention. Author’s own compilation. Legend: HiAP, Health is all Policies; SDH, Social deterninations of health; HIA, Health impact assessments; NCD, Non-communicable diseases.

Inspired by the Portuguese experience, we propose a CEIP-Framework (Figure 1) to emphasize the need to strengthen HiAP, namely when a national agenda is absent.

The CEIP-Framework considers comprehensive health legislation (C), enhanced reinforcement and monitoring (E), increased cross-sector collaboration (I), and public health investment (P).

Health legislation should prioritize health equity in policy-making, particularly in sectors that impact SDH [3], and integrate it into national, regional, and local health strategic plans [7].

Enhanced enforcement and monitoring are essential for improving health policy impact, including health impact assessments (HIA), and ensuring transparency [4]. Investment in data infrastructure, research and data sharing through record linkage is key to improving monitoring and addressing health inequalities [5, 9].

There is a need for formal HiAP guidance to align ministries with public health objectives, guide HIA, and foster cross-sector collaboration [4]. Involving stakeholders in advocacy and capacity-building promotes policy coherence and public acceptance [8].

Specific tax revenues should not be diverted from health programs to reduce the burden of non-communicable diseases (NCDs) [4]. Balancing revenue generation while protecting public health is key.

Regular evaluation and adaptation mechanisms must be implemented for effective framework outcomes, which are (Figure 1):

1. Health equity through integrated policies.
2. Preventive rather than reactive policies through enhanced monitoring and enforcement.
3. Cross-sector strengthened collaboration driving greater investment in public health, aligning relevant government and industry stakeholders, and enhancing the implementation of public health policies.
4. Reinvestment of revenues into primary prevention strategies.

This approach might help to better succeed in HiAP. Transformative approaches cannot fail to address the root causes of health inequalities and the rising burden of NCDs linked to key public health challenges: tobacco, sugar, alcohol and gambling.
