# Supplementary material for: The CEIP-Framework – From Reaction to Prevention in Health in All and for Policies
Source: Public Health Rev. 2025 Mar 21;46:1608225. doi: 10.3389/phrs.2025.1608225 (PMC11973516; doi:10.3389/phrs.2025.1608225)
Supplement: Supplementary file 1 [file DataSheet1.pdf]

Law references

[1] Law No. 95/2019 of September 4th.
Articles 18, 64, 168 and 198 of the Constitution of the Portuguese Republic (PRC).
Rules of Procedure of the Assembly of the Republic (RPAR) no. 1/2023, of 31 August.
Articles 29, 33, 34, 35,119, 124 § 1, paragraph c), § 2 and § 3, 130, 131, 135, 137, 138, 139, 142, 149, 150, 152 and 173 of the RPAR;
Decree-Law nº 32/2024 of May 10th.
Articles 14, paragraph a), Article 6, § 3, paragraphs b) and c), Article 22, 31, 33, 35, 36, 37 and 38 of The Rules of Procedure of the Council of Ministers (RPCM) approved by the Resolution of the Council of Ministers no. 65/2024, of April 24th.
Article 3 of the Legislative Rules for the Preparation of Government Normative Acts (LRPGNA) of the XIV Constitutional Government approved by the Resolution of the Council of Ministers no. 65/2024, of April 24th.
Resolution of the Council of Ministers no. 65/2024, of April 24th.
Law No. 98/2021 of December 31st.

[2] primarily governed by Law nr 37/2007

[3] in with the European Union Tobacco Products Directive

[4] Regulations under Decree-Law nr 50/2013

[5] (Decree-Law No. 73/2010, of June 21, which approved the Code of Special Consumption Taxes - Part II - Special part - Chapter I - Tax on beverages containing added sugar or other sweeteners).

[6] Regime Jurídico dos Jogos e Apostas Online (Legal Regime for Online Gaming and Betting, approved by the Decree-Law No. 66/2015, of April 29) and Gambling Law (approved by the Decree-Law No. 422/89 of December 2)
